# Impressive response to dabrafenib, trametinib, and osimertinib in a metastatic *EGFR-mutant*/*BRAF* V600E lung adenocarcinoma patient

**DOI:** 10.1038/s41698-021-00149-4

**Published:** 2021-02-12

**Authors:** Maurício Fernando Silva Almeida Ribeiro, Franciele Hinterholz Knebel, Fabiana Bettoni, Rodrigo Saddi, Karina Perez Sacardo, Felipe Sales Nogueira Amorim Canedo, João Victor Machado Alessi, Andrea Kazumi Shimada, José Flávio Gomes Marin, Anamaria Aranha Camargo, Artur Katz

**Affiliations:** 1 Oncology Center, Hospital Sírio-Libanês, Rua Dona Adma Jafet, 91, 01308-050 São Paulo, SP Brazil; 2 Molecular Oncology Center, Hospital Sírio-Libanês, Rua Dona Adma Jafet, 91, 01308-050 São Paulo, SP Brazil; 3 Nuclear Medicine Center, Hospital Sírio-Libanês, Rua Dona Adma Jafet, 91, 01308-050 São Paulo, SP Brazil

**Keywords:** Non-small-cell lung cancer, Molecular medicine, Biomarkers

## Abstract

The survival outcomes of the FLAURA trial support osimertinib as the new standard of care for untreated patients harboring activating mutations in the epidermal growth factor receptor (*EGFR*). Despite the initial response, disease progression invariably occurs. Although uncommon, *BRAF* V600E mutation arises as a unique mechanism of resistance, and thus far, no prospective studies are available to support concurrent *EGFR/BRAF* blockade. We report a case of impressive radiological and ctDNA response under dabrafenib, trametinib, and osimertinib in an advanced *EGFR-*mutant lung adenocarcinoma patient who developed *BRAF* V600E as one of the acquired resistance mechanisms to second-line osimertinib. Moreover, the patient experienced remarkable clinical improvement and good tolerance to combination therapy. The present case suggests the importance of prospective studies evaluating both efficacy and safety of the combination in later line settings and points towards the potential of ctDNA to monitor resistance mechanisms and treatment benefit in clinical practice.

## Introduction

The use of osimertinib to target epidermal growth factor receptor (EGFR) has become the standard of care in untreated *EGFR*-mutant non–small cell lung cancer (NSCLC) patients. Although osimertinib can be highly active, showing more durable outcomes than first-generation tyrosine kinase inhibitors (TKI)^1^, most tumors invariably become resistant, limiting its long-term clinical benefit. The heterogeneity of resistance mechanisms to osimertinib, including *EGFR* C797S mutation, *EGFR*, and *MET* amplifications, off-target mutations in *PIK3CA, KRAS*, and *HER2* as well as histologic transformation^2–9^, has stimulated routine performance of repeated biopsies to identify specific underlying mechanisms of resistance throughout the treatment course, and to guide the development of novel therapeutic strategies to overcome and prevent acquired resistance (AR)^7,10–13^.

Combined targeted therapy (TT) strategies have been increasingly addressed in prospective clinical trials^14–17^. A phase Ib/II trial reported 47% ORR with a combination of capmatinib (MET inhibitor) plus gefitinib in patients progressing to EGFR TKI and presenting *MET* dysregulation^14^. Likewise, SAVANNAH (NCT03778229)^15^ is an ongoing phase II trial designed to evaluate the efficacy of combination savolitinib and osimertinib in post-osimertinib progression/MET-positive patients. The biomarker-matched study ORCHARD (NCT03944772)^16^ is also underway to assess the efficacy of several osimertinib-based combinations following disease progression under frontline osimertinib.

*BRAF* mutations and fusions (i.e. *AGK-BRAF*, *ESYT2-BRAF*) have recently emerged as additional mechanisms of AR to third-generation EGFR TKI^2,18–20^. Studies demonstrating the efficacy of concurrent inhibition of EGFR and BRAF^3^ or MEK^21^ in pre-clinical models have raised clinicians’ expectations about overcoming AR by combining TT. Nevertheless, reports of successful combinations of TT for patients harboring *BRAF*-driven AR to osimertinib are very limited^19,22–24^ and no prospective data regarding efficacy and safety of BRAF/MEK/EGFR concurrent inhibition are available, with chemotherapy-based regimens remaining the treatment of choice in this unfavorable scenario. Similarly, *PIK3CA* mutations may also mediate AR to second-line osimertinib in 4–11%, but no clinical reports suggesting potential benefits of blocking these alterations in NSCLC are available^6,9,25–27^.

The detection of circulating tumor DNA (ctDNA) using liquid biopsies allows noninvasive real-time monitoring of treatment response and early detection of AR to TT, anticipating radiological response and treatment failure^28,29^. The widespread availability of highly specific and sensitive techniques to quantify ctDNA makes the longitudinal assessment of patients with NSCLC under TKI therapy very attractive. Nonetheless, for NSCLCs, ctDNA evaluation in routine clinical practice is currently limited to the detection of *EGFR* exon 19 deletion, *EGFR* L858R and T790M activating mutations^29^.

A man with metastatic lung adenocarcinoma harboring an *EGFR* mutation, who had progressed to erlotinib due to the emergence of the *EGFR* T790M mutation started osimertinib and remained on treatment for 15 months, then developing disease progression (PD). Sequential liquid biopsies were collected to monitor treatment response and disclosed the emergence of *BRAF* V600E and *PIK3CA* E545K resistance mutations 4 months before clinical progression. With this result and considering few available reports in the literature, our patient started a triple therapy with osimertinib, dabrafenib (BRAF inhibitor), and trametinib (MEK inhibitor).

## Results

### Case report

A 50-year-old non-smoker man was diagnosed with a tubule-papillary lung adenocarcinoma metastatic to bones and soft tissue (stage IVB - AJCC 8^th^ edition) in July 2016. Several hypermetabolic bone lesions were observed on staging 18-fluorodeoxyglucose positron-emission tomography (18F-FDG PET-CT) scan at diagnosis. A baseline Next Generation Sequencing (NGS) assay (TruSightTumor™ - Illumina®) of a soft tissue metastasis revealed the presence of an activating *EGFR* exon 19 deletion (*EGFR* E746_A750del). No concurrent alterations in *BRAF* or in *PIK3CA* were identified at that time. ALK d5f3 immunohistochemistry and fluorescent in situ hybridization (FISH) for *ROS1* and *RET* resulted negative.

**Fig. 1:**
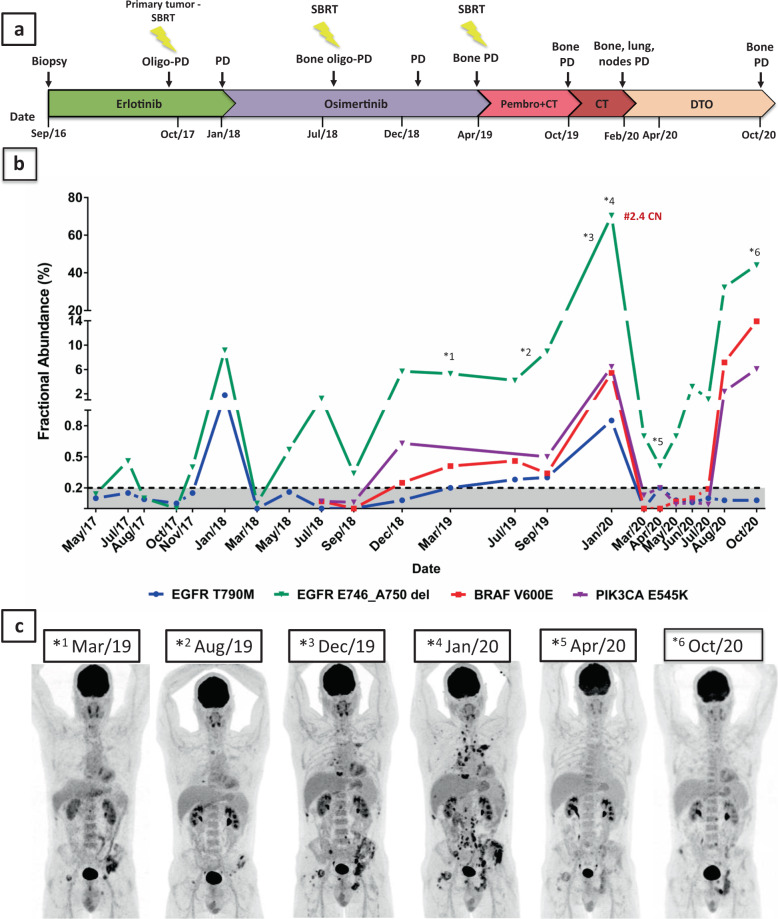
Paired radiological and sequential blood-based ctDNA assessments throughout patient’s treatment demonstrating concordant results. **a** Timeline displaying systemic and focal therapies since September 2016. **b** ctDNA levels in serial plasma samples based on fractional abundance of *EGFR* T790M, *EGFR* del19 (E746_A750 del), *BRAF* V600E, and *PIK3CA* E545K mutations. ^#^2.4 CN results: EGFR del19 WT/RNAseP copy number ratio. **c** Paired 18F-FDG PET-CT scan maximum intensity projection (MIP) images displaying tumor burden variations. PD progression of disease, SBRT stereotatic body radiotherapy, Pembro pembrolizumab, CT chemotherapy, DTO dabrafenib, trametinib and osimertinib.

In September 2016, the patient started on erlotinib 150 mg once daily (OD), achieving complete metabolic response in February 2017. We started to perform serial blood sample collections for ctDNA analysis in May 2017. In October 2017, oligo-progression (oligo-PD) was observed at the primary site and treated with stereotactic body radiation therapy (SBRT; 3 × 16 Gy). In November 2017, we identified the *EGFR* T790M and *EGFR* E746_A750del mutations in patient’s plasma using droplet digital PCR (ddPCR) and these results were confirmed in December 2017 with the Foundation ACT® ctDNA assay. Figure 1a shows all systemic and focal therapies of this patient since diagnosis.

Erlotinib was kept until January 2018 when disease progression in the bones was observed and the fractional abundance (FA) of *EGFR* E746_A750del and *EGFR* T790M were 12.0 and 2.3%, respectively (Fig. 1b). The treatment was promptly switched to osimertinib 80 mg OD. Bone partial response (PR) was observed 2 months later, along with a significant drop in the FA of *EGFR* E746_A750del and *EGFR* T790M mutations in the plasma (Fig. 1b).

In July 2018, the patient developed oligo-PD in T3 vertebrae, which was treated with SBRT (1 × 20 Gy). At that point, ctDNA analysis revealed an increase in the FA of the *EGFR* E746_A750del in the plasma, but not in *EGFR* T790M, suggesting genetic heterogeneity between metastatic lesions. In December 2018, a new oligo-PD in the left iliac bone was detected by 18F-FDG PET-CT scan (Fig. 1c). At that time, ctDNA analysis revealed the emergence of *BRAF* V600E (FA: 0.4%) and *PIK3CA* E545K (FA: 0.9%) mutations, as well as a significant increase in the FA of the *EGFR* E746_A750del (FA: 7.5%) in the plasma. These mutations were also detected in a specimen from an iliac bone biopsy using a NGS assay (TruSightTumor™ - Illumina® - *EGFR* del19 allelic fraction 81.1%, *EGFR* del19 amplification – 12 copies, *BRAF* V600E allelic fraction 17.7% and *PIK3CA* E545K allelic fraction 32.7%). Even though the patient was treated with SBRT (1 × 18 Gy), he developed new bone metastases in March 2019 (Fig. 1b), consistent with the significant increase in the FA of *EGFR* E746_A750del, *BRAF* V600E and *EGFR* T790M mutations (Fig. 1b). At that specific timepoint and in April 2019, assessments of *PIK3CA* E545K plasma levels were not obtained due to limited amount of cfDNA.

Between April and September 2019, systemic treatment with carboplatin plus pemetrexed and pembrolizumab provided modest clinical benefit, reducing tumor burden and controlling the disease (Fig. 1b). Due to a new PD observed in October 2019, the treatment was switched to docetaxel monotherapy and, subsequently, to vinorelbine after progression under docetaxel; nonetheless, the patient experienced symptomatic PD in bones, lymph nodes, and lung in late January 2020 (Fig. 1c, 2a–d). He came to the clinic with a Karnofsky performance status of 70%, complaining of fatigue, appetite loss, and severe pain in the hips (despite regular use of 10 mg buprenorphine patch and oxycodone plus acetaminophen PO), which had been preventing him from performing his daily activities due to the inability to stay seated. At that point, high levels of all three resistance mutations were detected in the circulating DNA (*PIK3CA* E545K FA: 13.2%, *BRAF* V600E FA: 12.3%, and *EGFR* T790M FA: 5.3%; Fig. 1b). We also observed an exponential increase in the number of copies of *EGFR* E746_A750del, suggesting gene amplification (Fig. 1b).

**Fig. 2:**
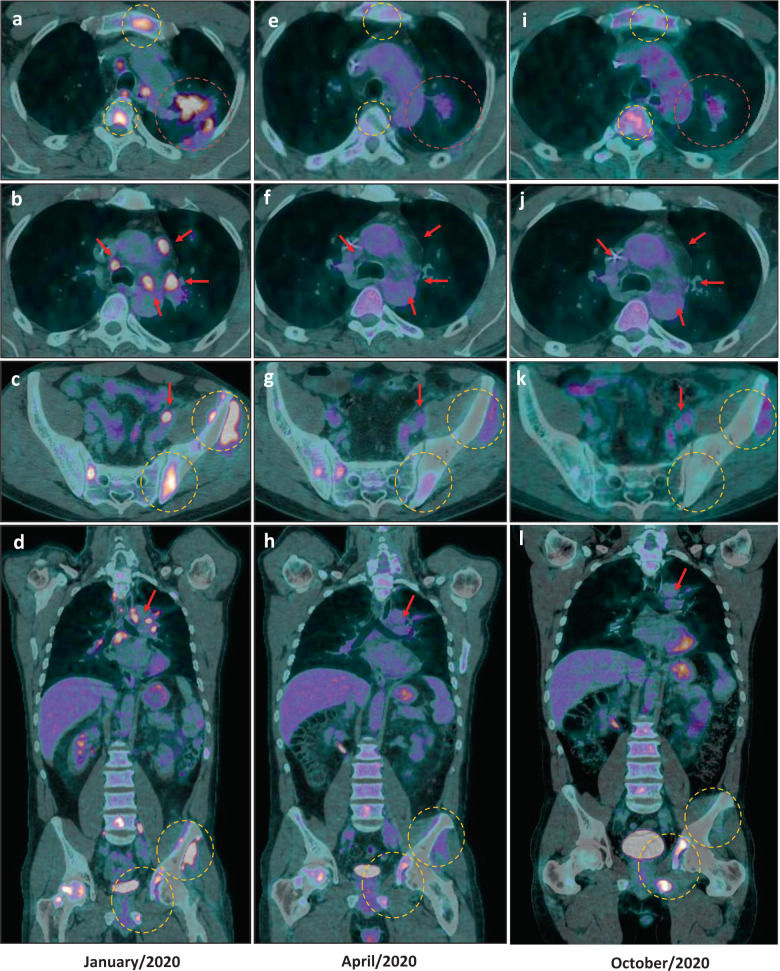
18F-FDG PET-CT scan imaging depicting impressive overall tumor response under dabrafenib, trametinib, and osimertinib in three timepoints. **a**–**d** baseline imaging (January/2020) showing: hypermetabolic spiculated mass (orange circle) in the left-superior lobe measuring 4.4 × 3.3 cm (SUVmax: 9.4); multiple hilar, mediastinal, retroperitoneal, and iliac hypermetabolic lymph nodes (red arrow) measuring up to 2.7 cm (SUVmax: 12.8); several hypermetabolic bone lesions throughout axial and appendicular skeleton (yellow circle); a left iliac bone lesion with signs of periosteal reaction and adjacent soft-tissue infiltration (SUVmax: 11.4). **e**–**h** first response evaluation imaging (April/2020) showing considerable partial response in the lung mass, measuring 3.8 × 2.5 cm (SUVmax: 2.7), as well as in several bone lesions, especially in the left-iliac bone (SUVmax: 4.8); complete response in lymph nodes. **i**–**l** third response evaluation imaging showing disease progression in the left ischium (SUVmax: 12.5; previous SUVmax: 5.9) (October/2020). SUVmax maximum standard uptake value.

In February 2020, after careful consideration due to patient’s good performance status, normal organ functions, severe pain, motivation and the emergence of *BRAF* V600E as an AR mechanism, he started on dabrafenib 75 mg twice daily (BID), trametinib 1 mg OD and osimertinib 80 mg OD. The use of a PI3K inhibitor was not considered an option due to the absence of reports in the literature suggesting efficacy of these drugs combined with osimertinib to treat advanced NSCLC patients. Within 2 weeks of treatment, the patient achieved complete resolution of the severe bone pain in the hips with no further need of opioid administration, as well as appetite gain and marked improvement in quality of life, which turned possible for him to resume his daily activities. As adverse events (AE), he experienced grade 1 fatigue, dysgeusia, fever, and nausea, all managed with symptomatic medication. Complete resolution of the fever occurred spontaneously within two weeks of treatment. An attempt to increase dosages of dabrafenib to 150 mg BID and trametinib 2 mg OD resulted unsuccessful due to persistent grade 2 fatigue. In April 2020, a new 18F-FDG PET-CT scan disclosed a complete response in lymph nodes and a dramatic PR in the lung and bones (PERCIST 1.1 evaluating 5 target lesions: Δ- 67%; Figs. 1c and 2e–h). Detection of *EGFR* T790M*, BRAF* V600E, and *PIK3CA* E545K mutations became negative in April 2020, while *EGFR* E746_A750del despite marginally positive, presented marked reduction (Fig. 1b) suggesting that combination dabrafenib, trametinib, and osimertinib might be an effective strategy to overcome *PIK3CA* E545K and *BRAF* V600E-driven resistance to osimertinib in advanced *EGFR*-mutant NSCLC patients. The patient remained in response until October 2020, when he developed asymptomatic bone PD in lumbar spine, left ischium, and right iliac bone, 8 months after starting this combination therapy (Figs. 1c and 2i–l). A FoundationOne®Liquid CDx plasma NGS disclosed the following alterations: *CHCHD3-BRAF* fusion, *BRAF* V600E, *EGFR* E746_750del, *EGFR* amplification, *PIK3CA* E545K, *MAP2K2* (*MEK2*) C125S, *MTAP* rearrangement intron 5, *TP53* V197M, and *TP53* S241A. Owing to the considerable clinical benefit, good tolerance and lack of systemic treatment options, we decided to keep the patient on treatment and increase the doses of dabrafenib (150 mg BID) and trametinib (2 mg OD) every other day. He remains asymptomatic and tolerating well the proposed dose adjustment.

## Discussion

Several studies have recently highlighted the importance of considering genes of interest within the context of commonly co-occurring mutations^9,30^. For example, as described by Blakely et al. through performing a cfDNA NGS analysis of 1,122 advanced stage *EGFR*-mutant NSCLCs, in ~93% of the patients, at least one more variables with known or likely known functional properties were present, disclosing the molecular complexity of this oncogenic driver and suggesting an association of co-occuring genomic alterations with TKI response and clinical outcomes^9^. In addition, Roper and colleagues reported the identification of at least two co-existing AR mechanisms in 73% of patients treated with osimertinib, as well as 6–23 different subclones per individual in a phylogenetic analysis performed in multiple metastatic sites of 15 individuals^30^. In the same publication, the authors also called the attention for a high incidence of acquired *EGFR* amplifications in post-osimertinib patients, which suggests maintenance of *EGFR* central role in the setting of progression, as previously reported by our group^28^ and also detected in the present case.

Although uncommon, *BRAF* V600E mediates AR in approximately 3% of the patients under second-line osimertinib^6,20^ and little is known about the efficacy of combined TT in this population^3,21–23^. Existing reports lack details regarding objective responses using standardized radiological criteria (i.e., RECIST 1.1; PERCIST) and the dynamics of resistance mechanisms through longitudinal ctDNA measurements^22,23^. Huang and colleagues^22^ described a case of an *EGFR*del19/T790M + NSCLC patient who developed *BRAF* V600E-driven AR after second-line osimertinib and achieved stable disease under dabrafenib 150 mg BID, trametinib 1 mg OD and osimertinib 80 mg OD, with ongoing disease control 7.4 months after. Zhou and colleagues^23^ also reported their experience with dabrafenib 150 mg BID, trametinib 2 mg OD and osimertinib 80 mg OD leading to tumor reduction within 6 weeks of treatment, along with grade 2 rash and decreased appetite as AEs. Similarly, Meng et al. reported two cases treated with this triple regimen^31^. The first patient discontinued therapy after one month due to severe pneumonitis; the second one presented tumor response under dabrafenib 50 mg BID, trametinib 0.5 mg OD, and osimertinib 80 mg OD, with progression-free survival of 14 months. According to the authors, this reduced dose was prescribed owing to a grade 2 pyrexia, nausea, and vomiting under higher dabrafenib and trametinib doses. Dagogo-Jack and colleagues^19^ also described a successful case of combined EGFR/MAP kinase pathway blockade with osimertinib 80 mg OD and trametinib 1 mg OD; as treatment-related AEs, their patient experienced grade 2 diarrhea and fatigue, along with grade 1 rash and gastrointestinal bleeding. Nevertheless, it is worth highlighting the rapid clinical improvement, the remarkable radiologically confirmed objective response, as well as the good tolerance observed in this case even using only half standard dose of dabrafenib and trametinib approved for NSCLCs harboring *BRAF* V600E mutations. Since data regarding the efficacy of these combined approaches, the optimal drug association and dosing, as well as the toxicity profile are conflicting and largely unknown, further investigation into the mechanistic basis of this association represents an important priority. The Table 1 summarizes the toxicities arising under osimertinib plus BRAF/MEK inhibition and reported in the literature.

**Table 1:** Summary of the toxicities arising under osimertinib plus BRAF/MEK inhibition and reported in the literature.

Reference	Treatment	Anorexia	Nausea	Vomiting	Diarrhoea	Fatigue	Rash	GI bleeding	AST/ALT elevation	Paroniquia	Pyrexia	Initial dose	Dose reduction
Meng et al.	D + T + O	NR	G2	G2	G2	NR	NR	NR	NR	NR	G2	D (150 mg BID) + T (2 mg OD) + O (80 mg OD)	D (50 mg BID) + T (0.5 mg/day) + O (80 mg/day)
Dagogo-Jack et al.	T + O	NR	NR	NR	G2	G2	G1	G1	G1	NR	NR	T (1 mg OD) + O (80 mg OD)	Not needed
Huang et al.	D + T + O	NR	NR	NR	G1	NR	NR	NR	NR	G1	NR	D (150 mg BID) + T (1 mg OD) + O (80 mg OD)	Not needed
Zhou et al.	D + T + O	G1	NR	NR	NR	NR	G2	NR	NR	NR	NR	D (150 mg BID) + T (2 mg OD) + O (80 mg OD)	Not needed

*D* dabrafenib, *T* trametinib, *O* osimertinib, *NR* not reported.

Liquid biopsy is emerging as an important diagnostic and predictive tool in the treatment of NSCLCs. The accurate identification of predictive genetic alterations is important for both patients’ management and the understanding of clonal evolution and AR to different therapies^9,29,32^. Also, circulating biomarkers from multiple disease sites better reflect systemic tumor burden, including alterations from genetically different metastatic lesions, which may be missed with single-site tissue biopsies^8^. Here, we were able to identify and monitor multiple concurrent mechanisms of resistance throughout the entire patient’s treatment. As shown in Fig. 1b, variations in blood ctDNA levels exhibited a positive correlation with imaging findings, even in situations involving focal treatments for oligo-PD. The observed concordance between radiological PD or response and ctDNA measurements also points towards its great potential to be incorporated into clinical practice to anticipate radiologic findings in a more effective manner.

Along with *BRAF* V600E mutation, the presence of *PIK3CA* E545K mutation is also associated with AR to osimertinib^6,9,25,27^. However, the clinical implication of concomitant targeting of EGFR and PI3K remains unclear. Whereas an alternative argument for the observed response could be a rechallenge following a long time upon off-osimertinib, in our opinion, this is an unlikely explanation^33^. Interestingly, the presence of a *BRAF* fusion along with a *MEK2* mutation identified through plasma NGS in the setting of PD might represent a mechanism of resistance to this triple regimen^34^. Nonetheless, since pretreatment blood samples had not been analyzed using a similar methodology, this hypothesis deserves careful interpretation.

Increased understanding of the relationship of concurring genomic alterations in *EGFR*-mutant NSCLC may enable new therapeutic opportunities following disease progression to osimertinib. Here, we reported an impressive objective response to dabrafenib, trametinib, and osimertinib with concordant decrease in plasma ctDNA levels in a metastatic lung cancer patient harboring *EGFR* E746_A750del, *BRAF* V600E, and *PIK3CA* E545K activating mutations. This case report leads to a greater understanding of the currently limited literature regarding the management of *EGFR*-mutant NSCLC patients with acquired *BRAF*V600E mutation, since it reports a successful attempt to target both alterations concurrently while providing concordant and interesting data of serial ctDNA assessments throughout the entire treatment. Further investigation to optimize the efficacy and mitigate the toxicity profile of this drug association represents an important issue.

## Methods

### Patient

This study was approved by Hospital Sírio-Libanês Ethics Committee (HSL-RC 2020-16). The patient provided written informed consent for blood collection, ctDNA analysis and publication of this report.

### Sample collection and plasma DNA extraction

Serial blood samples were collected between May 2017 and April 2020 (Fig. 1b). Peripheral blood (20 ml) was collected into tubes containing EDTA (BD, Franklin Lakes, New Jersey). Plasma was separated from the blood within 2 hours of blood collection, as previously described^28^. cfDNA was extracted using QIAamp MinElute Virus Vacuum Kit (Qiagen, Hilden, Germany) and stored at −80 °C.

### ctDNA-ddPCR

Cell-free DNA (cfDNA) was quantified using the RNase P Copy Number Reference Assay (Life Technologies, Carlsbad, California). A total of 3000 genome-equivalents (~10 ng of cfDNA) were analyzed per assay to achieve a detection sensitivity of 0.2%. This detection limit has been assessed by using cell line-derived genomic DNA. A total of 10 ng of input DNA with varying proportions of mutant DNA was serially diluted into wild-type DNA to obtain samples with a mutant abundance of 1%, 0.5%, 0.1%, and 0.05% and subjected to droplet digital PCR (ddPCR). ddPCR was used to quantify the circulating levels of the EGFR activating mutation (*EGFR* E746_A750 del) and of the resistance mutations (*EGFR* T790M, *PIK3CA* E545K, and *BRAF* V600E). Probes and primers were obtained from BioRad (*EGFR* E746_A750 del #10041170, *EGFR* T790M #10040782, *PIK3CA* E545K #10041188, and *BRAF* V600E #10040779; Hercules, California). ddPCR was performed on the QX200 Droplet Digital PCR System, and data were analysed using QuantaSoft software (Bio-Rad). ctDNA quantification is presented as fractional abundance (FA–the proportion of the mutant allele in total cfDNA).

### Reporting summary

Further information on research design is available in the Nature Research Reporting Summary linked to this article.

## Supplementary information

Reporting Summary

Dataset 1

## Data Availability

The datasets that support the findings of this study are not publicly available in order to protect patient privacy. The data will be made available on reasonable request. For data access requests regarding the liquid biopsy (ctDNA quantification) data, please contact Dr. Franciele Knebel, email address: fhknebel@mochsl.org.br. For data access requests regarding the PET-CT high resolution images and PERCIST calculations, please contact Dr. José Marin, email address:jfgmarin@yahoo.com.br. For data access requests regarding the summary of the toxicities arising under osimertinib plus BRAF/MEK inhibition, please contact the corresponding author Dr. Maurício Ribeiro, email address: mauricio.fsaribeiro@hsl.org.br. The data generated and analysed during this study are described in the following metadata record: 10.6084/m9.figshare.13475946^35^.
